# Development of off-the-shelf hematopoietic stem cell-engineered invariant natural killer T cells for COVID-19 therapeutic intervention

**DOI:** 10.1186/s13287-022-02787-2

**Published:** 2022-03-21

**Authors:** Yan-Ruide Li, Zachary Spencer Dunn, Gustavo Garcia, Camille Carmona, Yang Zhou, Derek Lee, Jiaji Yu, Jie Huang, Jocelyn T. Kim, Vaithilingaraja Arumugaswami, Pin Wang, Lili Yang

**Affiliations:** 1 Department of Microbiology, Immunology and Molecular Genetics, University of California, Los Angeles, Los Angeles, CA 90095 USA; 2 Mork Family Department of Chemical Engineering and Materials Science, University of Southern California, Los Angeles, Los Angeles, CA 90089 USA; 3 Department of Molecular and Medical Pharmacology, University of California, Los Angeles, Los Angeles, CA 90095 USA; 4 Division of Infectious Diseases, Department of Medicine, University of California, Los Angeles, Los Angeles, CA 90095 USA; 5 Eli and Edythe Broad Center of Regenerative Medicine and Stem Cell Research, University of California, Los Angeles, Los Angeles, CA 90095 USA; 6 Jonsson Comprehensive Cancer Center, David Geffen School of Medicine, University of California, Los Angeles, Los Angeles, CA 90095 USA; 7 Molecular Biology Institute, University of California, Los Angeles, Los Angeles, CA 90095 USA

**Keywords:** Hematopoietic stem cell (HSC), Invariant natural killer T (iNKT) cell, Coronavirus disease 2019 (COVID-19), Severe acute respiratory syndrome coronavirus 2 (SARS-CoV-2), Allogeneic adoptive cell transfer, Off-the-shelf cellular product

## Abstract

**Background:**

New COVID-19 treatments are desperately needed as case numbers continue to rise and emergent strains threaten vaccine efficacy. Cell therapy has revolutionized cancer treatment and holds much promise in combatting infectious disease, including COVID-19. Invariant natural killer T (iNKT) cells are a rare subset of T cells with potent antiviral and immunoregulatory functions and an excellent safety profile. Current iNKT cell strategies are hindered by the extremely low presence of iNKT cells, and we have developed a platform to overcome this critical limitation.

**Methods:**

We produced allogeneic HSC-engineered iNKT (^Allo^HSC-iNKT) cells through TCR engineering of human cord blood CD34^+^ hematopoietic stem cells (HSCs) and differentiation of these HSCs into iNKT cells in an Ex Vivo HSC-Derived iNKT Cell Culture. We then established in vitro SARS-CoV-2 infection assays to assess ^Allo^HSC-iNKT cell antiviral and anti-hyperinflammation functions. Lastly, using in vitro and in vivo preclinical models, we evaluated ^Allo^HSC-iNKT cell safety and immunogenicity for off-the-shelf application.

**Results:**

We reliably generated ^Allo^HSC-iNKT cells at high-yield and of high-purity; these resulting cells closely resembled endogenous human iNKT cells in phenotypes and functionalities. In cell culture, ^Allo^HSC-iNKT cells directly killed SARS-CoV-2 infected cells and also selectively eliminated SARS-CoV-2 infection-stimulated inflammatory monocytes. In an in vitro mixed lymphocyte reaction (MLR) assay and an NSG mouse xenograft model, ^Allo^HSC-iNKT cells were resistant to T cell-mediated alloreaction and did not cause GvHD.

**Conclusions:**

Here, we report a method to robustly produce therapeutic levels of ^Allo^HSC-iNKT cells. Preclinical studies showed that these ^Allo^HSC-iNKT cells closely resembled endogenous human iNKT cells, could reduce SARS-CoV-2 virus infection load and mitigate virus infection-induced hyperinflammation, and meanwhile were free of GvHD-risk and resistant to T cell-mediated allorejection. These results support the development of ^Allo^HSC-iNKT cells as a promising off-the-shelf cell product for treating COVID-19; such a cell product has the potential to target the new emerging SARS-CoV-2 variants as well as the future new emerging viruses.

**Supplementary Information:**

The online version contains supplementary material available at 10.1186/s13287-022-02787-2.

## Introduction

SARS-CoV-2, the etiologic agent of the COVID-19 pandemic, is responsible for over 40 million cases and 700 thousand deaths in the United States alone [1]. As case numbers continue to rise, there are increasing concerns that COVID-19 may stay/recur within human society for an extended period [2], and that vaccines, although highly effective and produced with unprecedented speed [3–5], may not be adequate to end COVID-19 [6] Positive cases in vaccine recipients can occur [7], emergent strains may evade memory responses [8], and for several reasons significant proportions of society are unvaccinated [9]. Despite tremendous efforts to generate antiviral drugs and therapeutic interventions, including nucleoside analogs [10, 11], chloroquine [10], protease inhibitors [12], monoclonal antibody therapy [13–15], and cell-based therapy [16, 17], only remdesivir (Veklury) has received United States Food and Drug Administration (FDA) approval for treating COVID-19, notwithstanding an absence of survival benefit [18]. Several other drugs, including sotrovimab (Xevudy), the antibody cocktail of casirivimab and imdevimab (Ronapreve), and tocilizumab (Actemra), have received FDA Emergency Use Authorizations, highlighting the potential of biologics to treat COVID-19. To further broaden the treatment options available for COVID-19 patients, novel therapies are urgently needed.

Cell-based immunotherapy has reshaped cancer treatment [19–21] and shown strong clinical efficacy in the treatment of infectious disease [22–24], and is now being investigated for COVID-19 [16, 17]. A recent study of critically ill COVID-19 patients reported the functional alteration of innate T cells, including invariant natural killer T (iNKT) and mucosal associated invariant T (MAIT) cells, showing that the patients contained significantly reduced numbers of iNKT cells and the activation status of iNKT cells was predictive of disease severity, suggesting the involvement of iNKT cells in COVID-19 [25]. iNKT cells are a unique subpopulation of T cells expressing a canonical invariant TCR α chain (Vα24-Jα18 in human) complexed with a semi-variant TCR β chain (mainly Vβ11 in human) that recognizes lipid antigens presented by CD1d, a non-polymorphic MHC Class I-like protein [26]. These cells play an important role in linking innate and adaptive immune responses and have been implicated in infectious disease, allergy, asthma, autoimmunity, and tumor surveillance [26–28]. A growing body of work indicates that iNKT cells play a beneficial role in battling acute respiratory virus infection, as these cells were shown to boost early innate immune responses, reduce viral titer, and inhibit the suppressive capacity of myeloid-derived suppressor cells (MDSCs) to enhance virus-specific responses in influenza models [28–32]. iNKT cells also reduced the accumulation of inflammatory monocytes in the lungs and decreased immunopathology during severe influenza A virus infection [29], demonstrating the potential for iNKT cells to have dual antiviral functions by direct virus clearance and inflammatory monocyte regulation. Importantly, because iNKT cells do not recognize mismatched MHC molecules and protein autoantigens, they are not expected to cause graft-versus-host disease (GvHD) [33–35]. This notion is strongly supported by clinical data analyzing donor-derived iNKT cells in blood cancer patients receiving allogeneic bone marrow or peripheral blood stem cell transplantation. These clinical data showed that the levels of engrafted allogenic iNKT cells in patients correlated positively with graft-versus-leukemia effects and negatively with GvHD [33–35]. Multivariate analyses of different immune cell subsets in allogeneic grafts showed that iNKT cell dose was the only graft parameter associated with a significant improvement in GvHD [36]. Therefore, iNKT cells are considered to be suitable for developing allogeneic “off-the-shelf” cell therapy targeting COVID-19.

Current iNKT cell-based therapies are restricted by the extremely low number and high variability of iNKT cells in peripheral blood [37, 38]. To overcome this critical limitation, we genetically engineered hematopoietic stem cells (HSCs) to express the iNKT TCR, which engendered the in vivo lineage commitment and expansion of both mouse and human HSC engineered iNKT (HSC-iNKT) cells following bone marrow transfer [39, 40]. However, such an in vivo approach can only be translated for autologous HSC adoptive therapies [40]. Recently, we have developed an Ex Vivo HSC-Derived iNKT Cell Culture method that can robustly produce therapeutic levels of allogeneic HSC-engineered human iNKT (^Allo^HSC-iNKT) cells. Here, we report the preclinical study of an ^Allo^HSC-iNKT cell therapy, showing its feasibility, safety, and COVID-19 therapy potential.

## Methods

### Lentiviral vectors and transduction

The lentivector and lentivirus were generated as previously described [40]. Lentiviral vectors used in this study were all constructed from a parental lentivector pMNDW. The Lenti/iNKT-sr39TK vector was constructed by inserting into pMNDW a synthetic tricistronic gene encoding human iNKT TCRa-F2A-TCRb-P2A-sr39TK; the Lenti/FG vector was constructed by inserting into pMNDW a synthetic bicistronic gene encoding Fluc-P2A-EGFP; the Lenti/ACE2 vector was constructed by inserting into pMNDW a synthetic gene encoding human ACE2. The synthetic gene fragments were obtained from GenScript and IDT. Lentiviruses were generated using 293T cells, following a standard calcium precipitation protocol and an ultracentrifigation concentration protocol or a tandem tangential flow filtration concentration protocol as previously described [39].

### SARS-CoV-2 virus generation

SARS-CoV-2, isolate USA-WA1/2020, was obtained from the Biodefense and Emerging Infections (BEI) Resources of the National Institute of Allergy and Infectious Diseases. All procedures involving SARS-CoV-2 infection were conducted within a Biosafety Level 3 facility at UCLA with appropriate institutional biosafety approvals. SARS-CoV-2 was passaged in African green monkey kidney epithelial cells (Vero E6; CRL-1586), which were maintained in D10 media comprised of Dulbecco’s modified Eagle’s medium (DMEM) supplemented with 10% fetal bovine serum (FBS; Omega Scientific) and 1% penicillin/streptomycin (P/S; Gibco). Viral stocks from the P6 passage were aliquoted and stored at − 80 °C for this study. To assess viral titers, Vero E6 cells were infected and examined daily for cytopathic effect (CPE). TCID50 was calculated based on the method of Reed and Muench [41].

### Antibodies and flow cytometry

All flow cytometry stains were performed in PBS for 15 min at 4 °C. The samples were stained with Fixable Viability Dye eFluor506 (e506) mixed with Mouse Fc Block (anti-mouse CD16/32) or Human Fc Receptor Blocking Solution (TrueStain FcX) prior to antibody staining. Antibody staining was performed at a dilution according to the manufacturer’s instructions. Fluorochrome-conjugated antibodies specific for human CD45 (Clone H130), TCRαβ (Clone I26), CD4 (Clone OKT4), CD8 (Clone SK1), CD45RO (Clone UCHL1), CD161 (Clone HP-3G10), CD69 (Clone FN50), CD56 (Clone HCD56), CD62L (Clone DREG-56), CD14 (Clone HCD14), CD1d (Clone 51.1), NKG2D (Clone 1D11), DNAM-1 (Clone 11A8), IFN-γ (Clone B27), granzyme B (Clone QA16A02), perforin (Clone dG9), β2-microglobulin (B2M) (Clone 2M2), HLA-DR (Clone L243) were purchased from BioLegend; Fluorochrome-conjugated antibodies specific for human CD34 (Clone 581) and TCR Vɑ24-Jβ18 (Clone 6B11) were purchased from BD Biosciences. Unconjugated human ACE2 antibody was purchased from R&D Systems. Fluorochrome-conjugated Donkey anti-goat IgG was purchased from Abcam. Human Fc Receptor Blocking Solution (TrueStain FcX) was purchased from BioLegend, and Mouse Fc Block (anti-mouse CD16/32) was purchased from BD Biosciences. Fixable Viability Dye e506 were purchased from Affymetrix eBioscience. Intracellular cytokines were stained using a Cell Fixation/Permeabilization Kit (BD Biosciences). Flow cytometry were performed using a MACSQuant Analyzer 10 flow cytometer (Miltenyi Biotech) and data analyzed with FlowJo software version 9.

### ***In vitr***o generation of allogeneic HSC-engineered iNKT (^Allo^HSC-iNKT) cells

Frozen-thawed human CD34^+^ HSCs were revived in HSC-culture medium composed of X-VIVO 15 Serum-free Hematopoietic Cell Medium supplemented with SCF (50 ng/ml), FLT3-L (50 ng/ml), TPO (50 ng/ml), and IL-3 (10 ng/ml) for 24 h; the cells were then transduced with Lenti/iNKT-sr39TK viruses for another 24 h following an established protocol [40]. The transduced HSCs were then collected and cultured ex vivo to differentiate into iNKT cells, via an Artificial Thymic Organoid (ATO) culture or a Feeder-Free culture. In the ATO culture, transduced HSCs were mixed with MS5-DLL4 feeder cells to form ATOs and cultured over ~ 8 weeks following a previously established protocol [42, 43]. In the Feeder-Free culture, transduced HSCs were cultured using a StemSpan™ T Cell Generation Kit (StemCell Technologies) over ~ 5 weeks following the manufacturer’s instructions. Differentiated ^Allo^HSC-iNKT cells were then collected and expanded with αGC-loaded PBMCs for 1–2 weeks following a previously established protocol [39]. The resulting ^Allo^HSC-iNKT cell products were collected and cryopreserved for future use.

### Generation of PBMC-derived conventional αβT, and iNKT cells

Healthy donor PBMCs were provided by the UCLA/CFAR Virology Core Laboratory and were used to generate the PBMC-Tc and PBMC-iNKT cells.

To generate PBMC-Tc cells, PBMCs were stimulated with CD3/CD28 T-activator beads (ThermoFisher Scientific) and cultured in C10 medium supplemented with human IL-2 (20 ng/mL) for 2–3 weeks, following the manufacturer’s instructions.

To generate PBMC-iNKT cells, PBMCs were MACS-sorted via anti-iNKT microbeads (Miltenyi Biotech) labeling to enrich iNKT cells, which were then stimulated with donor-matched irradiated αGC-PBMCs at the ratio of 1:1, and cultured in C10 medium supplemented with human IL-7 (10 ng/ml) and IL-15 (10 ng/ml) for 2–3 weeks. If needed, the resulting PBMC-iNKT cells could be further purified using Fluorescence-Activated Cell Sorting (FACS) via human iNKT TCR antibody (Clone 6B11; BD Biosciences) staining.

### Cell phenotype and functional study

Phenotype and functionality of multiple types of cells were analyzed, including ^Allo^HSC-iNKT, PBMC-iNKT, and PBMC-Tc cells. Phenotype of these cells was studied using flow cytometry, by analyzing cell surface markers including co-receptors (CD4 and CD8), NK cell markers (CD161), memory T cell markers (CD45RO), and NK activating receptors (NKG2D and DNAM-1). Capacity of cells to produce cytokine (IFN-γ) and cytotoxic factors (Perforin and Granzyme B) was measured using Cell Fixation/Permeabilization Kit (BD Biosciences). PBMC-Tc and PBMC-iNKT cells were included as FACS analysis controls.

### SARS-CoV-2 infection

SARS-CoV-2 infection was performed as previously described [44]. For SARS-CoV-2 infection, viral inoculum (MOI of 0.1 and 1) was prepared using serum-free medium. Culture medium was removed and replaced with 250 μL of prepared inoculum in each well. For mock infection, serum-free medium (250 μL/well) was added. The inoculated plates were incubated at 37 °C with 5% CO2 for 1 h. The inoculum was spread by gently tilting the plate sideways at every 15 min. At the end of incubation, the inoculum was replaced with fresh medium.

### In vitro killing assay of SARS-CoV-2 infected target cells

293T-FG, 293T-ACE2-FG, or Calu3-FG target cells (1 × 10^3^ cells per well) were seeded in Corning 96-well clear bottom black plates in D10 medium at day 0. At day 1, viral inoculum (MOI of 0.01) was prepared using D10 media. Media was gently removed without disrupting cells and replaced with 100 μl of prepared viral inoculum. ^Allo^HSC-iNKT cells (1 × 10^4^ cells per well) were then added in the plates at day 2. At day 3 or day 4, live target cells were detected by using Luciferase Assay System (CAT #E1500, Promega) following its protocol. Medium was carefully removed from the wells, and 1 × lysis reagent was added (20 μl per well) to lyse tumor cells and inactivate SARS-CoV-2 virus. Then the cell lysate was mixed with 50 μl of Luciferase Assay Reagent, and the luciferase activities were immediately analyzed using an Infinite M1000 microplate reader (Tecan). In the blocking assay, 10 μg per ml of LEAF™ purified anti-human NKG2D (Clone 1D11, Biolegend), anti-human DNAM-1 antibody (Clone 11A8, Biolegend), or LEAF™ purified mouse lgG2b κ isotype control antibody (Clone MG2B-57, Biolegend) was added to cell cultures when adding ^Allo^HSC-iNKT cells at day 2.

### Enzyme-linked immunosorbent cytokine assays (ELISA)

The ELISA for detecting human cytokines were performed following a standard protocol from BD Biosciences. Supernatants from co-culture assays were collected, mixed with equal-volume 0.02% Triton™ X-100 reagent (Sigma-Aldrich), and assayed to quantify IFN-γ. Triton™ X-100 reagent was utilized for inactivating SARS-CoV-2 viruses. The capture and biotinylated pairs for detecting cytokines were purchased from BD Biosciences. The streptavidin-HRP conjugate was purchased from Invitrogen. Human cytokine standards were purchased from eBioscience. Tetramethylbenzidine (TMB) substrate was purchased from KPL. The samples were analyzed for absorbance at 450 nm using an Infinite M1000 microplate reader (Tecan).

### Immunofluorescence imaging

293T-FG, 293T-ACE2-FG, or Calu3-FG target cells (2 × 10^3^ cells per well) were seeded in Chamber Slides in D10 medium at day 0. SARS-CoV-2 viral inoculum were added in the plates at day 1. ^Allo^HSC-iNKT cells (2 × 10^4^ cells per well) were added at day 2. At day 4, supernatant was carefully removed. Cells were fixed in 4% paraformaldehyde (PFA) for 15 min, washed with PBS, followed by permeabilization and blocking in blocking buffer (PBS containing 0.1% Triton X-100 and 5% donkey serum) for 1 h at room temperature. Primary antibodies were diluted in blocking buffer and incubated with cells at 4 °C for overnight. The next day, cells were washed with PBS and incubated with secondary antibodies for 1 h at room temperature. Secondary antibodies were diluted in 1 × PBS at 1:500 dilution. After incubation, cells were washed with PBS, incubated with DAPI (1:10,000) for 15 min, and mounted with Fluoromount-G reagent. The primary antibodies used include mouse anti CD3, 1:500 and mouse anti SARS-CoV-2 Spike, 1:200.

### In vitro mixed lymphocyte reaction (MLR) assay: studying elimination of SARS-CoV-2 infection promoted inflammatory monocytes

293T-FG, 293T-ACE2-FG, or Calu3-FG target cells (1 × 10^3^ cells per well) were seeded in Corning 96-well clear bottom black plates in D10 medium at day 0. At day 1, viral inoculum (MOI of 0.01) was prepared using D10 media. Media was gently removed without disrupting cells and replaced with 100 μl of prepared viral inoculum. ^Allo^HSC-iNKT cells (1 × 10^4^ cells per well) and donor-mismatched PBMCs were (1 × 10^4^ cells per well) were added in the plates at day 2. At day 3 or day 4, cells were analyzed by using flow cytometry. The culture supernatant was carefully removed from the wells, flow antibodies were added into the cells and incubated for 15 min on ice, and then the stained cells were fixed by 4% PFA for 1 h. 4% PFA was also used here to inactivate SARS-CoV-2. Then flow cytometry was used to analyze the cell numbers and phenotypes. In the blocking assay, 10 μg per ml of LEAF™ purified anti-human CD1d (Clone 51.1, Biolegend), or LEAF™ purified mouse lgG2b κ isotype control antibody (Clone MG2B-57, Biolegend) was added to cell cultures at day 2.

### In vitro mixed lymphocyte reaction (MLR) assay: studying graft-versus-host (GvH) response

PBMCs of multiple healthy donors were irradiated at 2500 rads and used as stimulators, to study the GvH response of ^Allo^HSC-iNKT cells as responders. PBMC-Tc cells were included as a responder control. Stimulators (5 × 10^5^ cells/well) and responders (2 × 10^4^ cells/well) were co-cultured in 96-well round bottom plates in C10 medium for 4 days; the cell culture supernatants were then collected to measure IFN-γ production using ELISA.

### In vitro MLR assay: studying host-versus-graft (HvG) response

PBMCs of multiple healthy donors were used as responders, to study the HvG response of ^Allo^HSC-iNKT cells as stimulators (irradiated at 2500 rads). PBMC-Tc cells were included as a stimulator control. Stimulators (5 × 10^5^ cells/well) and responders (2 × 10^4^ cells/well) were co-cultured in 96-well round bottom plates in C10 medium for 4 days; the cell culture supernatants were then collected to measure IFN-γ production using ELISA.

### GvH response of ^Allo^HSC-iNKT cells in human NSG mouse model

NSG mice (6–10 weeks of age) were pre-conditioned with 100 rads of total body irradiation, and then injected with 1 × 10^7 Allo^HSC-iNKT cells or donor-matched PBMCs intravenously. Over time, mouse survival rates were recorded.

### Statistical analysis

GraphPad Prism 6 (Graphpad Software) was used for statistical data analysis. Student’s two-tailed *t* test was used for pairwise comparisons. Ordinary 1-way ANOVA followed by Tukey’s multiple comparisons test was used for multiple comparisons. Log rank (Mantel-Cox) test adjusted for multiple comparisons was used for Meier survival curves analysis. Data are presented as the mean ± SEM, unless otherwise indicated. In all figures and figure legends, “n” represents the number of samples or animals utilized in the indicated experiments. A P value of less than 0.05 was considered significant. ns, not significant; *P < 0.05; **P < 0.01; ***P < 0.001; ****P < 0.0001.

## Results

### Generation of allogeneic HSC-engineered iNKT (^Allo^HSC-iNKT) cells

**Fig. 1:**
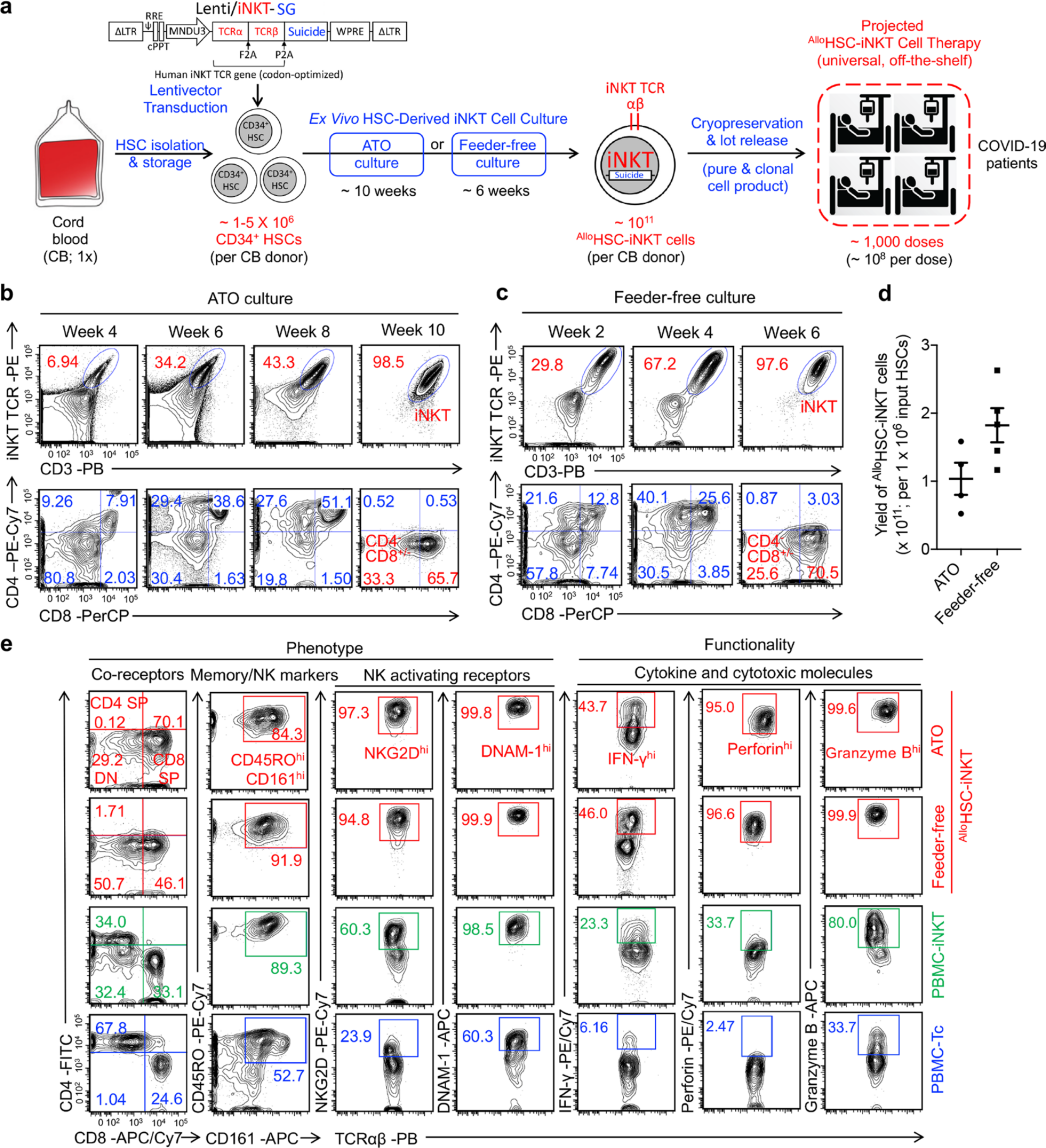
In vitro generation and characterization of allogenic HSC-engineered iNKT (^Allo^HSC-iNKT) cells. **a** Experimental design to generate ^Allo^HSC-iNKT cells in vitro. *CB* cord blood, *HSC* hematopoietic stem cell, *SG* suicide gene, *Lenti/iNKT-SG* lentiviral vector encoding an iNKT TCR gene and a suicide gene, *ATO* artificial thymic organoid. **b** Generation of iNKT cells (identified as iNKT TCR^+^CD3^+^ cells) during ATO culture. A 6B11 monoclonal antibody was used to stain iNKT TCR. **c** Generation of iNKT cells (identified as iNKT TCR^+^CD3^+^ cells) during Feeder-free culture. **d** Yields of ^Allo^HSC-iNKT cells generated from ATO and Feeder-free cultures. **e** FACS characterization of surface marker expression and intracellular cytokine and cytotoxic molecule production of ^Allo^HSC-iNKT cells. Periphery blood mononuclear cell (PBMC)-derived iNKT (PBMC-iNKT) cells and conventional αβ T (PBMC-Tc) cells were included as controls. Representative of over 5 experiments

Human cord blood (CB) CD34^+^ hematopoietic stem and progenitor cells (denoted as HSCs) were transduced with a Lenti/iNKT-SG vector and then differentiated into iNKT cells in an Ex Vivo HSC-Derived iNKT (HSC-iNKT) cell culture, using either an Artificial Thymic Organoid (ATO) culture system or a Feeder-Free culture system (Fig. 1a). The Lenti/iNKT-SG vector has been previously used to generate autologous HSC-engineered iNKT cells for cancer immunotherapy [40]; Depending on the needs, in the same lentivectors we can include a suicide gene (SG) (e.g., sr39TK) to provide cell products with a “safety switch” [40]; ATO is 3D cell culture system supporting the ex vivo differentiation of human T cells from HSCs [42, 43]; the Feeder-Free culture adopts a system of plate-bound delta-like ligand 4 (DLL4) and vascular cell adhesion protein 1 (VCAM-1) to enable T lymphoid differentiation [45–48]. Lentivector transduced HSCs were seeded in ATO culture or Feeder-free culture, where HSCs differentiated into human iNKT cells over a course of 10 weeks or 6 weeks, respectively, resulting in greater than 97% pure and clonal ^Allo^HSC-iNKT cells without bystander conventional αβ T cells (Fig. 1a–c). During the Ex Vivo HSC-derived iNKT cell cultures, ^Allo^HSC-iNKT cells followed a typical iNKT cell development path defined by CD4/CD8 co-receptor expression [37]. ^Allo^HSC-iNKT cells transitioned from CD4^−^CD8^−^ to CD4^+^CD8^+^, then to CD4^−^CD8^+/−^ (Fig. 1b, c). At the end of cultures, over 95% of the ^Allo^HSC-iNKT cells demonstrated a CD4^−^CD8^+/−^ phenotype (Fig. 1b, c).

The manufacturing process of generating ^Allo^HSC-iNKT cells using either ATO culture or Feeder-free culture were robust and of high yield and high purity for all CB donor tested (Fig. 1d). Based on the results, it was estimated that from one single CB donor (comprising ~ 1–5 × 10^6^ HSCs), ~ 10^11 Allo^HSC-iNKT cells could be generated that can potentially be formulated into ~ 1000 doses (Fig. 1d) [49–52]. The ^Allo^HSC-iNKT cell products contained pure transgenic iNKT cells and nearly undetectable bystander conventional αβ T cells, therefore reducing GvHD risk and supporting the use of ^Allo^HSC-iNKT cells as an off-the-shelf therapy.

### Phenotype and functionality of ^Allo^HSC-iNKT cells

To study their phenotype and functionality, we compared ^Allo^HSC-iNKT cells to healthy donor periphery blood mononuclear cell (PBMC)-derived iNKTs and conventional αβ T cells (denoted as PBMC-iNKT and PBMC-Tc cells, respectively). Both ATO and Feeder-Free cultured ^Allo^HSC-iNKT cells displayed typical iNKT cell phenotype similar to that of PBMC-iNKT cells, but distinct from that of PBMC-Tc cells. ^Allo^HSC-iNKT cells presented as CD4^−^CD8^+/−^ cells and expressed high levels of memory T cell marker CD45RO and NK cell marker CD161 (Fig. 1e). In addition, compared to PBMC-iNKT and PBMC-Tc cells, ^Allo^HSC-iNKT cells upregulated NK activating receptors like NKG2D and DNAM-1 and produced exceedingly high levels of the effector cytokine IFN-γ and cytotoxic molecules like Perforin and Granzyme B (Fig. 1e), indicating the potent effector potential of ^Allo^HSC-iNKT cells. For example, the percentage of ^Allo^HSC-iNKT cells identified by flow cytometry as expressing high levels of IFN-γ (45%) was double that of PBMC-Tc cells (23%). When stimulated with αGC, which is a synthetic agonist glycolipid antigen specifically stimulating iNKT cells [26], ^Allo^HSC-iNKT cells proliferated vigorously (Additional file 1: Fig. S1a, c) and secreted high levels of Th0/Th1 cytokines like IFN-γ, TNF-α, and IL-2, while limited levels of Th2 cytokine IL-4 and Th17 cytokine IL-17 (Additional file 1: Fig. S1b, d), showing a Th0/Th1-prone functionality of ^Allo^HSC-iNKT cells. Despite the manufacturing difference, ^Allo^HSC-iNKT cells generated from ATO culture or Feeder-free culture displayed similar phenotype and functionality (Fig. 1e; Additional file 1: S1a–d); in this report, these cells were alternatively used and showed comparable COVID-19 targeting potential.

### Direct killing of SARS-CoV-2-infected cells by ^Allo^HSC-iNKT cells

**Fig. 2:**
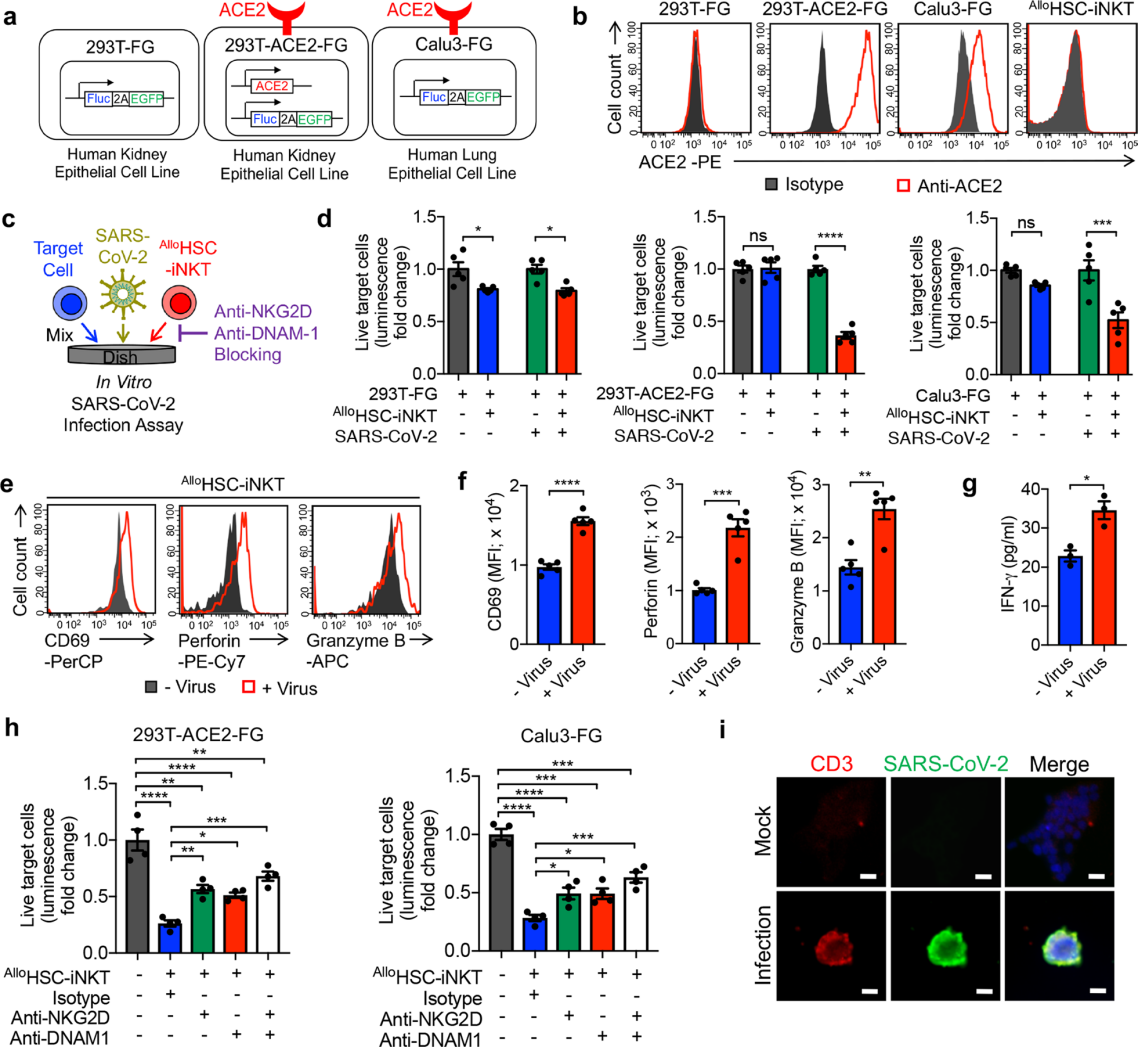
^Allo^HSC-iNKT cells directly target and kill SARS-CoV-2 infected cells. **a** Schematics showing the engineered 293T-FG, 293T-ACE2-FG, and Calu3-FG cell lines. *ACE2* angiotensin converting enzyme 2, *Fluc* firefly luciferase, *EGFP* enhanced green fluorescent protein, *F2A* foot-and-mouth disease virus 2A self-cleavage sequence. **b** FACS detection of ACE2 on 293T-FG, 293T-ACE2-FG, Calu3-FG, and ^Allo^HSC-iNKT cells. **c**–**h** In vitro direct killing of SARS-CoV-2 infected cells by ATO culture-generated ^Allo^HSC-iNKT cells. **c** Experimental design. **d** Target cell killing data of ^Allo^HSC-iNKT cells at 24-h post co-culturing with infected cells (*n* = 5). **e** FACS detection of CD69, Perforin and Granzyme B of ^Allo^HSC-iNKT cells at 24-h post co-culturing with SARS-CoV-2 infected 293T-ACE2-FG cells. **f** ELISA analysis of IFN-γ production (*n* = 3). **h** SARS-CoV-2 infected cell killing mechanisms of ^Allo^HSC-iNKT cells. NKG2D and DNAM-1 mediated pathways were studied (*n* = 5). **i** Immunofluorescence analysis of direct targeting of SARS-CoV-2 infected cells by ^Allo^HSC-iNKT cells. Representative of 3 experiments. Data are presented as the mean ± SEM. ns, not significant, *P < 0.05, **P < 0.01, ***P < 0.001, ****P < 0.0001, by Student's *t* test (**d**, **f** and **g**), or by 1-way ANOVA **h**. See also Additional file 1: Fig. S1

Following the successful generation of ^Allo^HSC-iNKT cells, we established an in vitro SARS-CoV-2 infection assay to assess the direct killing of SARS-CoV-2-infected cells. Studies have indicated that SARS-CoV-2 can infect multiple tissues in addition to lung tissue [53, 54]. We therefore established in vitro infection models using two cell lines: a human kidney epithelial cell line, 293T, and a human lung epithelial cell line, Calu-3 (Fig. 2a, b). The parental 293T cell line does not express ACE2, and we engineered a subline to overexpress ACE2 (Fig. 2b). All target cell lines were also engineered to express a firefly luciferase (Fluc) and green fluorescence protein (EGFP) dual-reporters (Fig. 2b). As a result, three target cell lines were generated, 293T-FG, 293T-ACE2-FG, and Calu3-FG, with 293T-FG serving as a negative control for studying SARS-CoV-2 infection (Fig. 2a, b). Notably, ^Allo^HSC-iNKT cells do not express ACE2, indicating that these therapeutic cells themselves are not susceptible to SARS-CoV-2 infection (Fig. 2b). ^Allo^HSC-iNKT cells selectively killed 293T-ACE2-FG and Calu3-FG cells, but not the 293T-FG control cells, under SARS-CoV-2 infection conditions. This suggests that ^Allo^HSC-iNKT cells may have the potential to target SARS-CoV-2 infected cells without damaging uninfected tissue (Fig. 2c, d; Additional file 2: S2a, b). The killing of virus-infected target cells was associated with the activation of ^Allo^HSC-iNKT cells, as shown by their upregulated expression of activation markers (i.e., CD69) and production of effector molecules (i.e., Perforin, Granzyme B, and IFN-γ) (Fig. 2e–g; Additional file 3: S3a, b). In addition, the target cell killing was significantly reduced by blocking NK activation receptors, as the administration of anti-NKG2D or anti-DNAM1 antibodies into infection assays doubled the survival rate of 293T-ACE2-FG and Calu3-FG cells cultured with ^Allo^HSC-iNKT cells (Fig. 2h). Corroborating the cytotoxicity towards virus-infected cells, immunohistology imaging studies showed the selective clustering of ^Allo^HSC-iNKT cells with SARS-CoV-2-infected cells (Fig. 2i). Overall, ^Allo^HSC-iNKT cells demonstrated a potent capacity of direct killing of virus-infected cells and thereby may contribute to virus clearance.

### Elimination of virus-infection promoted inflammatory monocytes by ^Allo^HSC-iNKT cells

**Fig. 3:**
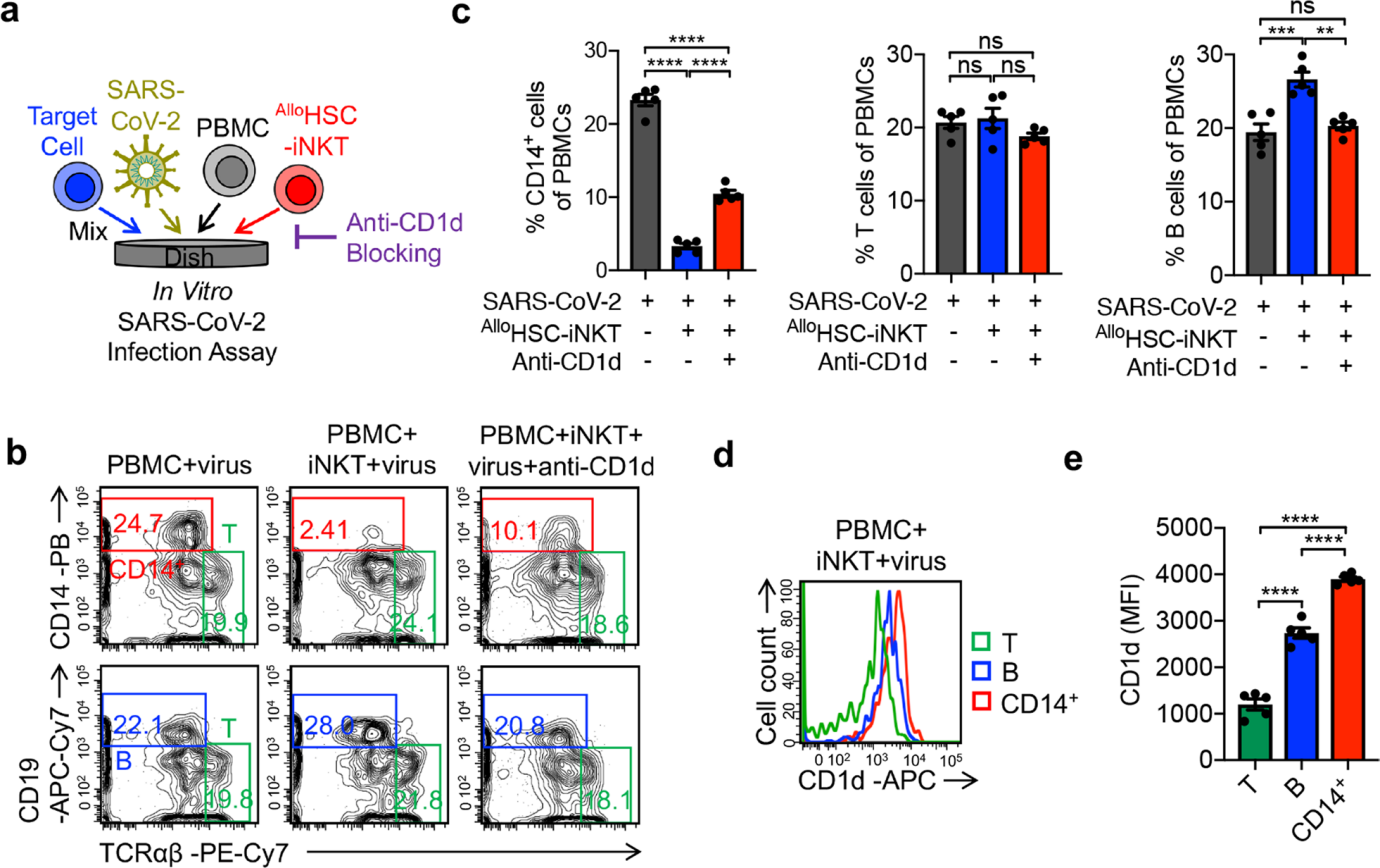
^Allo^HSC-iNKT cells reduce virus-infection promoted inflammatory monocytes. **a** Experimental design. 293T-ACE2-FG cells were infected by SARS-CoV-2 virus. After 1 day, ATO culture-generated ^Allo^HSC-iNKT cells and donor-mismatched PBMCs were added and incubated for 24 h. Flow cytometry was used to detect cell populations. **b** FACS detection of CD14^+^ monocytes, T cells, and B cells in PBMCs. **c** Quantification of **b** (*n* = 5). **d** FACS detection of CD1d expression on CD14^+^ monocytes, T cells, and B cells. **e** Quantification of **d** (*n* = 5). Representative of 3 experiments. Data are presented as the mean ± SEM. ns, not significant, *P < 0.05, **P < 0.01, ***P < 0.001, ****P < 0.0001, by 1-way ANOVA

Previous studies have reported that iNKT cells could reduce accumulation of inflammatory monocytes in the lungs and decrease immunopathology under virus infection [28, 29]. Therefore, we established another in vitro SARS-CoV-2 infection assay to study the elimination of virus-infection promoted inflammatory monocytes by ^Allo^HSC-iNKT cells via iNKT TCR/CD1d recognition (Fig. 3a). ^Allo^HSC-iNKT cells effectively eliminated CD14^+^ inflammatory monocytes under SARS-CoV-2 infection condition, an effect that was reduced in half by blocking CD1d (Fig. 3a–c; Additional file 2: S2c, d). Meanwhile, T cells and B cells in the same cultures were not impacted, suggesting that ^Allo^HSC-iNKT therapeutic cells may not compromise the T cell and B cell antiviral immunity (Fig. 3b, c; Additional file 2: S2c, d) [53, 54]. In agreement with an iNKT TCR/CD1d recognition-mediated mechanism, in the SARS-CoV-2 infection culture, inflammatory CD14^+^ monocytes expressed significantly higher levels of CD1d than that of T cells and B cells (Fig. 3d, e) [28, 29]. The anti-CD1d antibody did not completely block the CD14^+^ monocyte elimination (Fig. 3b, c), suggesting that there are other iNKT/monocyte recognition pathways remaining to be investigated. Interestingly, compared to the CD14^+^ monocytes in the assay without the addition of ^Allo^HSC-iNKT cells, the residual CD14^+^ monocytes after co-culturing with ^Allo^HSC-iNKT cells demonstrated a significant reduction in CD1d and CD86 expression levels (Additional file 4: Fig. S4). One possible reason was that ^Allo^HSC-iNKT cells targeted inflammatory CD14^+^ monocytes via iNKT TCR/CD1d recognition and CD28/CD86 co-stimulation. Virus infection promoted the upregulation of CD1d and CD86 on monocytes [55, 56], and the CD1d^high^CD86^high^ monocytes were easier to be targeted and eliminated by ^Allo^HSC-iNKT cells, resulting in a residual CD1d^low^CD86^low^ population (Additional file 4: Fig. S4). Overall, ^Allo^HSC-iNKT cells can potentially limit inflammation-mediated damage caused by virus infection by eliminating inflammatory monocytes [29].

### Safety study of ^Allo^HSC-iNKT cells

**Fig. 4:**
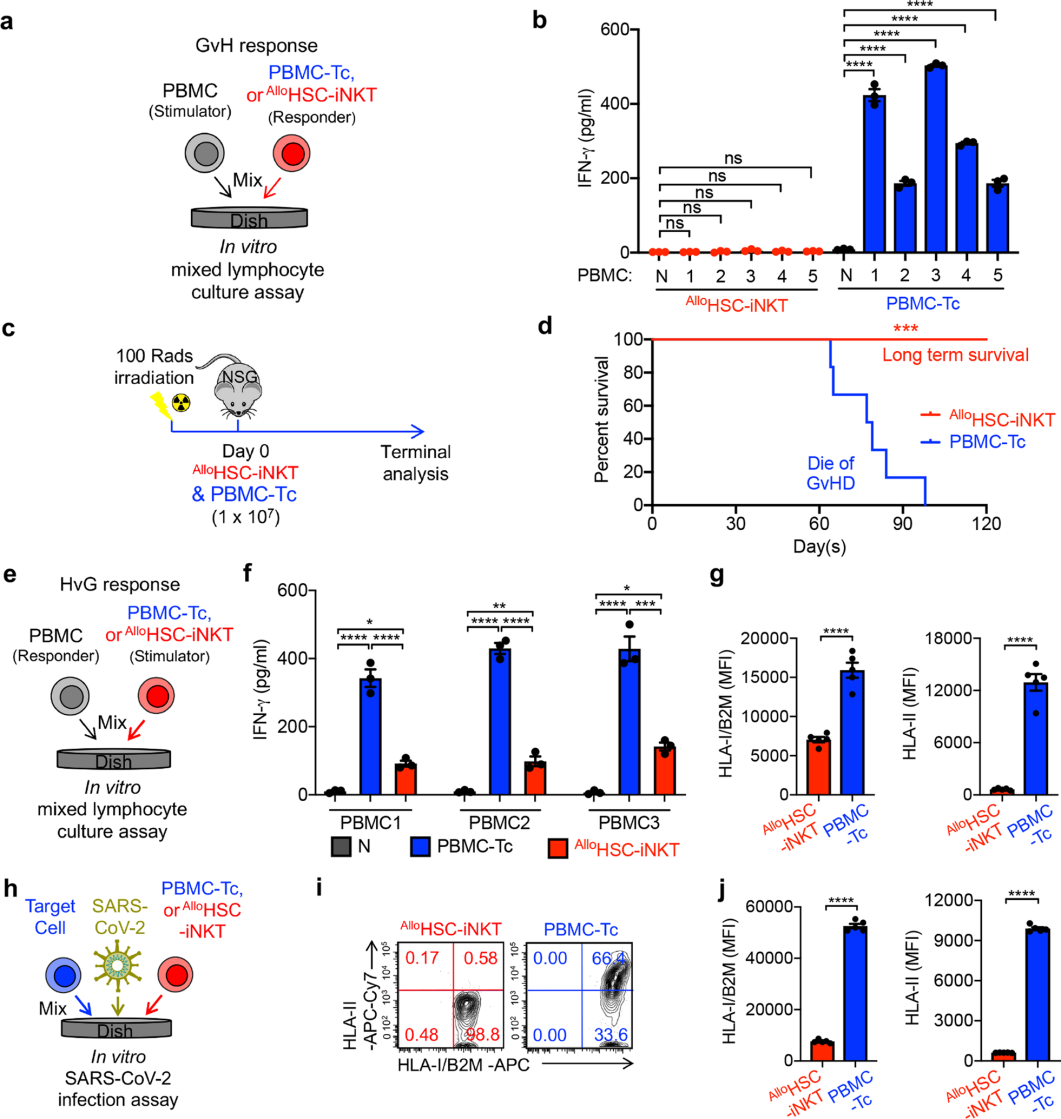
Safety and immunogenecity of ^Allo^HSC-iNKT cells. **a**–**b** Studying the graft-verus-host (GvH) response of ^Allo^HSC-iNKT cells using an in vitro mixed lymphocyte reaction (MLR) assay. PBMC-Tc cells were included as a responder cell control. **a** Experimental design. PBMCs from 5 different healthy donors were used as stimulator cells. **b** ELISA analyses of IFN-γ production at day 4 (*n* = 3). N, no stimulator cells. **c**–**d** Studying the GvH response of ^Allo^HSC-iNKT cells using NSG mouse model. PBMC-Tc were included as a control. **A** Experimental design. **B** Kaplan–Meier survival curves of experimental mice over time (*n* = 6). **e**–**g** Studying T cell-mediated alloreaction against ^Allo^HSC-iNKT cells using an in vitro MLR assay. Irradiated ^Allo^HSC-iNKT cells (as stimulators) were co-cultured with donor-mismatched PBMC cells (as responders). Irradiated PBMC-Tc cells were included as a stimulator cell control. **e** Experimental design. PBMCs from 3 different healthy donors were used as responders. **f** ELISA analyses of IFN-γ production at day 4 (*n* = 3). **g** FACS analyses of HLA-I and HLA-II expression on the indicated stimulator cells (*n* = 5). **h**–**j** Studying HLA-I and HLA-II expression on ^Allo^HSC-iNKT cells under SARS-CoV-2 infection. ^Allo^HSC-iNKT cells were co-cultured with SARS-CoV-2 infected target cells. PBMC-Tc cells were included as a control. **h** Experimental design. **i** FACS analyses of HLA-I and HLA-II expression on the indicated stimulator cells. **j** Quantification of **i** (*n* = 5). Representative of 3 experiments. Data are presented as the mean ± SEM. ns, not significant, *P < 0.05, **P < 0.01, ***P < 0.001, ****P < 0.0001, by Student's *t* test (**j** and **g**), by 1-way ANOVA (**b** and **f**), or by log rank (Mantel–Cox) test adjusted for multiple comparisons (**d**)

Graft-versus-host (GvH) response is a main safety concern for “off-the-shelf” allogeneic cell therapies [57]. Due to the expression of an invariant TCR targeting glycolipids presented by monomorphic MHC Class I-like CD1d molecules, iNKT cells do not react with mismatched HLA molecules and protein autoantigens, and are thus not expected to cause GvH response [33, 35]. We used an established in vitro Mixed Lymphocyte Reaction (MLR) assay and an in vivo NSG mouse xenograft model to study the GvH response of ^Allo^HSC-iNKT cells (Fig. 4a, c). In contrast to conventional PBMC-Tc cells, ^Allo^HSC-iNKT cells did not produce GvH responses against multiple mismatched-donor PBMCs, evidenced by their lack of IFN-γ secretion (Fig. 4b). In vivo*,* ^Allo^HSC-iNKT cell-treated experimental mice did not have GvHD and sustained long-term survival over 120 days, whereas PBMC-Tc cell-treated mice died of serious GvHD around two months post PBMC-Tc cell transfer (Fig. 4c, d). In vitro and in vivo, ^Allo^HSC-iNKT cells did not display GvHD risk.

### Immunogenicity study of ^Allo^HSC-iNKT cells

For allogeneic cell products, immunogenicity is another concern due to potential allorejection by host T cells [58]. Host conventional CD8 and CD4 αβ T cells target allogeneic cells through recognizing mismatched HLA-I and HLA-II molecules, respectively, and can greatly decrease the efficacy of therapeutic allogeneic cells [59, 60]. In an in vitro MLR assay studying T cell-mediated host-versus-graft (HvG), ^Allo^HSC-iNKT cells elicited significantly less IFN-γ secretion, a surrogate for HvG response, compared to PBMC-Tc cells (Fig. 4e, f). Flow cytometry analysis showed that ^Allo^HSC-iNKT cells expressed significantly reduced levels of HLA-I molecules than PBMC-Tc cells and nearly undetectable HLA-II molecules, indicating potential mechanisms for their resistance to T cell-mediated HvG responses (Fig. 4g). Because a virus infection-induced inflammatory microenvironment may upregulate the expression of HLA molecules on immune cells (e.g., via IFN-γ) [61], we also analyzed HLA expression on ^Allo^HSC-iNKT cells in the presence of SARS-CoV-2 infection (Fig. 4h). As shown by flow cytometry analysis, under virus infection conditions ^Allo^HSC-iNKT cells maintained low expressions of HLA-I and HLA-II molecules (Fig. 4i, j). Cumulatively, these studies demonstrated the stable, low level expression of HLA-I and HLA-II molecules on ^Allo^HSC-iNKT cells that may grant them resistance to host T cell-mediated rejection. The high safety and low immunogenicity features of ^Allo^HSC-iNKT cells greatly support their potential application for “off-the-shelf” cell therapy.

## Discussion

The case and death tolls due to SARS-CoV-2 infection continue to rise as we enter what appears to be another wave of COVID-19 [1]. The rapid, landmark development of highly effective vaccines [3–5] forms a crucial line of defense against COVID-19, but significant portions of society, for medical, accessibility, and other reasons, are unvaccinated [9]. Additionally, breakthrough cases occur [7], emergent virus strains threaten vaccine efficacy [8, 62], and the duration of protection engendered by infection or vaccination is unknown [9]. An off-the-shelf, variant-agnostic COVID-19 intervention is urgently needed to reduce patient mortality and protect vulnerable populations, and to provide a crucial window for the distribution of vaccines and potential subsequent doses [63].

Severe COVID-19 usually begins about one week after the onset of symptoms, and often manifests clinically as progressive respiratory failure that develops soon after dyspnea and hypoxemia [64, 65]. These patients commonly suffer acute respiratory distress syndrome (ARDS), and can also experience lymphopenia, thromboembolic complications, disorders of the central or peripheral nervous system, acute cardiac, kidney, and liver injury, shock, and death [64, 65]. The resulting organ failures correlate with signs of inflammation, including high fevers, heightened levels of proinflammatory cytokines and chemokines (i.e. IL-6, IL-8, MCP-1, CRP), and abnormal myeloid cell expansion and lung infiltration [66–68]. Thousands of clinical COVID-19 trials testing antiviral compounds, immunomodulators, neutralizing agents, combination therapies, and other therapies have been initiated [69]. While remdesirvir (Veklury), casirivimab/imdevimab (Ronapreve), and several other drugs have received FDA authorization, research into novel therapeutic strategies can further improve the COVID-19 treatment landscape and expand the antiviral armamentarium used to fight future emerging viruses.

Cell-based immunotherapies have recently transformed the clinical landscape of blood malignancies [50, 70–72] and are an active area of research for antiviral treatments, including COVID-19 [16, 17]. Invariant natural killer T (iNKT) are a rare, unique subpopulation of T cells that target lipid-based antigens presented by monomorphic MHC Class I-like CD1d molecules and have potent immunoregulatory functions [26–28]. iNKT cell therapy has proven safe with signs of clinical activity in combatting cancer [73], and accumulating evidence suggests iNKT cells can ameliorate respiratory viral infection [28, 74, 75]. In a model of mild influenza virus (IAV) infection, activation of iNKT cells reduced viral titers in the lungs of mice without affecting T cell immunity and was accompanied by a better disease course with improved weight loss profile [30]. Using models of lethal, high pathogenicity influenza infection, Santo et. al. and Kok et. al. demonstrated that the absence of iNKT cells in mice during IAV infection resulted in the expansion of myeloid cells and correlated with increased viral titer, lung injury, and mortality. Activation or adoptive transfer of iNKT cells abolished the suppressive activity of myeloid cells, restored influenza-specific immune responses, reduced IAV titer, and increased survival rate, and the crosstalk between iNKT and myeloid cells was CD1d-dependent [29, 31]. The results were extended to humans by demonstrating that the suppressive activity of myeloid cells present in the peripheral blood of individuals infected with influenza was substantially reduced by iNKT cell activation [31]. In another preclinical mouse model, Paget et. al. showed that iNKT cells limit influenza pathology in a preclinical mouse model through the production of IL-22 [32]. Importantly, a recent publication reporting on critically ill COVID-19 patients showed that the patients contained significantly reduced numbers of iNKT cells and the activation status of iNKT cells was predictive of disease severity, suggesting the involvement of iNKT cells in COVID-19 [25]. Collectively, these studies indicate that iNKT cells play an important and beneficial role in battling acute respiratory virus infection, through mediating virus clearance and supporting effector responses while also limiting the degree of lung injury by regulating other immune responses and virus-mediated sequelae.

A critical bottleneck in the clinical application of iNKT cells is their scarcity, as iNKT cells make up ~ 0.001–1% of peripheral blood cells. Two years ago, we reported the in vivo production of iNKT cells through TCR engineering of hematopoietic stem cells followed by bone marrow transfer. Advances in the Ex Vivo HSC-iNKT differentiation culture methods have allowed us to mature our platform into completely in vitro, CMC compliant systems that generate large quantities of pure, clonal ^Allo^HSC-iNKT cells. Characterization of ^Allo^HSC-iNKT cells revealed phenotypic and functional profiles comparable to endogenous peripheral blood iNKT cells, although ^Allo^HSC-iNKT cells were predominated (97%) double negative (DN, CD4^−^CD8^−^) or CD8 single positive (SP). Mouse iNKT cells are CD4^+^ SP or DN, whereas human iNKT cells are CD4^+^ SP, CD8^+^ SP, or DN. In mice and human, CD4^+^ SP iNKT cells display a Th2 phenotype, favoring IL-4 production, whereas DN iNKT in mice and CD8^+^ SP and DN iNKT cells in humans are Th1-like and produce large quantities of IFN-γ. Of note, when assessed for CD4 expression, CD4^−^ and CD4^+^ iNKT cells were present in equal proportions in influenza-infected lungs but only CD4^−^ iNKT cells exhibited cytotoxicity towards inflammatory monocytes [29]. In general, CD8^+^ SP/DN human iNKT cells are considered to be proinflammatory (Th0/Th1) and highly cytotoxic, while CD4^+^ SP human iNKT cells are considered to exhibit a more complex regulatory function [37, 76–78]. Therefore, the in vitro generated ^Allo^HSC-iNKT cells, which display a dominant CD8^+^ SP/DN phenotype, seem fit for application in both cancer therapy and virus infection treatment, although the biological regulations leading to this phenotype remain to be determined.

Our previous studies have demonstrated the HSC-derived iNKT cells could attack tumors through multiple mechanisms, including (1) direct killing of CD1d + tumor cells through iNKT TCR, (2) direct killing of tumor cells through NK activating receptors, (3) adjuvant effects on enhancing NK-mediated killing of tumor cells, (4) adjuvant effects on enhancing dendritic cell and cytotoxic T lymphocyte-mediated antitumor activities, and (5) inhibition of tumor-associated macrophages [40, 77, 78]. In this study, using in vitro models, we have gathered preliminary evidence supporting the usage of ^Allo^HSC-iNKT cells against SARS-CoV-2 infection. Firstly, ^Allo^HSC-iNKT cells lysed SARS-CoV-2-infected lung epithelial cells. Mechanistic analysis revealed NK-pathway mediated killing of SARS-CoV-2-infected cells, as the blocking of NKG2D or DNAM-1 receptors reduced target cell lysis (Fig. 2). In addition to the direct effect ^Allo^HSC-iNKT cells can have on SARS-CoV-2 replication, ^Allo^HSC-iNKT cells selectively eliminated virus-activated inflammatory monocytes. In the presence of SARS-CoV-2 infection, ^Allo^HSC-iNKT cells lysed monocytes, without affecting T cell or B cell populations, in a CD1d-influenced manner (Fig. 3). The results suggest that ^Allo^HSC-iNKT therapeutic cells will not compromise the T cell and B cell antiviral immunity important for combating COVID-19.

A major concern for allogeneic T cell-based therapies is GvHD [79], a potentially life-threatening disease in which donor T cells attack host tissue [80]. By targeting non-polymorphic CD1d, iNKT cells avoid causing GvHD and have displayed an excellent safety profile in the clinic [73]. Using mixed lymphocyte reactions and a preclinical mouse model, we did not observe GvH responses by ^Allo^HSC-iNKT cells, whereas PBMC-derived T cells secreted IFN-γ in vitro and caused lethal GvHD in vivo (Fig. 4a–d). It is also important that allogeneic cell therapies resist rejection by the host (i.e. HvG responses) to exert their therapeutic functions [79]. ^Allo^HSC-iNKT cells express remarkably low amounts of HLA-I and HLA-II molecules and maintained low expression of HLA-I and HLA-II molecules under SARS-CoV-2 infection (Fig. 4e–j). Accordingly, in vitro MLRs showed that ^Allo^HSC-iNKT cells are resistant to T cell-mediated allorejection.

Future studies testing iNKT cells in 3D human lung organoid infection models [81] and preclinical COVID-19 models will provide invaluable insights into the clinical application of ^Allo^HSC-iNKT cells. Two potential in vivo models are a human lung xenograft NSG mouse infection model [82] and a hACE2 transgenic mouse infection model [83]. The transgenic model will not support the direct study of human ^Allo^HSC-iNKT cells due to xeno-incompatibility, and we plan to generate mouse HSC-engineered iNKT (mHSC-iNKT) cells as a therapeutic surrogate. Previously, we successfully generated mouse HSC-iNKT cells and showed that they closely resemble their native counterparts [39].

Our work underscores the potential for using iNKT cells to combat COVID-19, specifically TCR-engineered, HSC-derived iNKT cells. Using an Ex Vivo culture method, we generated thousands of ^Allo^HSC-iNKT cell therapy doses from one cord blood donor. We hypothesize that ^Allo^HSC-iNKT cells can reduce SARS-CoV-2 pathogenicity through two distinct mechanisms: (1) direct killing of SARS-CoV-2 infected cells; (2) selective elimination of virus-activated inflammatory monocytes. Furthermore, our studies indicate ^Allo^HSC-iNKT cells do not exhibit graft-versus-host responses and are resistant to immune cell allorejection, indicating ^Allo^HSC-iNKT cells may be a promising “off-the-shelf” anti-COVID-19 therapy.

## Supplementary Information

**Additional file 1. Fig. S1**: Antigen responses of ^Allo^HSC-iNKT cells; Related to Fig. 1. ^Allo^HSC-iNKT cells generated from ATO and Feeder-free systems were cultured for 2 weeks, in the presence or absence of αGC (denoted as αGC or Vehicle, respectively). (a and c) Cell growth curve (n = 3). (b and d) ELISA analyses of cytokine (IFN-γ, TNF-α, IL-2, IL-4 and IL-17) production at day 7 post αGC stimulation (n = 3).

**Additional file 2. Fig. S2**: Reduction of SARS-CoV-2 Virus Infection Load and Virus Infection-Induced Hyperinflammation by Feeder-Free Culture-Generated ^Allo^HSC-iNKT Cells; Related to Fig. 2 and 3. (a-b) Study directly targeting of SARS-CoV-2 infected cells by Feeder-free culture-generated ^Allo^HSC-iNKT cells. (a) Experimental design. (b) Target cell killing data of ^Allo^HSC-iNKT cells at 24-h post co-culturing with infected cells (n = 5). (c-d) Study targeting virus-infection promoted inflammatory monocytes by Feeder-free culture-generated ^Allo^HSC-iNKT cells. (c) Experimental design. (d) Flow cytometry analysis of remaining CD14^+^ monocytes, T and B cells in PBMCs after co-culturing with ^Allo^HSC-iNKT cells. Representative of 3 experiments. Data are presented as the mean ± SEM. ns, not significant, *P < 0.05, **P < 0.01, ***P < 0.001, ****P < 0.0001, by Student's t test (b) and 1-way ANOVA (d).

**Additional file 3. Fig. S3**: ^Allo^HSC-iNKT Cells are Activated by SARS-CoV-2 Infected Target Cells; Related to Fig. 2. (a) FACS detection of CD69, Perforin, and Granzyme B of ^Allo^HSC-iNKT cells at 24-h post co-culturing with SARS-CoV-2 infected Calu3-FG cells. (b) Quantification of (a) (n = 5). (c) ELISA analysis of IFN-γ production (n = 3). Representative of 3 experiments. Data are presented as the mean ± SEM. ns, not significant, *P < 0.05, **P < 0.01, ***P < 0.001, ****P < 0.0001, by Student's t test.

**Additional file 4. Fig. S4**: Phenotype changes of CD14^+^ monocytes after co-culturing with ^Allo^HSC-iNKT cells under SARS-CoV-2 infection; Related to Fig. 3. 293T-ACE2-FG cells were infected by SARS-CoV-2 virus. After 1 day, PBMCs were seeded in the culture with or without the addition of ^Allo^HSC-iNKT cells and cultured for 24 h. Flow cytometry was used to detect cell populations. (a-b) FACS analyses of CD1d expression on CD14^+^ monocytes (n = 5). (c-d) FACS analyses of CD86 expression on CD14^+^ monocytes (n = 5). Data are presented as the mean ± SEM. **P < 0.01, by Student's t test.

## Data Availability

All data associated with this study are present in the paper or Supplemental information.

## Abbreviations

HSC: Hematopoietic stem cell
iNKT: Invariant natural killer T
COVID-19: Coronavirus disease 2019
SARS-CoV-2: Severe acute respiratory syndrome coronavirus 2
^Allo^HSC-iNKT: Allogeneic HSC-Engineered iNKT
GvHD: Graft-versus-host disease
HvG: Host-versus-graft
MLR: Mixed lymphocyte reaction
MAIT: Mucosal associated invariant T
MDSC: Myeloid-derived suppressor cell
TCR: T cell receptor
CB: Cord blood
PBMC: Peripheral blood mononuclear cell
SG: Suicide gene
ATO: Artificial thymic organoid
DLL4: Delta-like ligand 4
VCAM-1: Vascular cell adhesion protein 1
Fluc: Firefly luciferase
EGFP: Green fluorescence protein
F2A: Foot-and-mouth disease virus 2A self-cleavage sequence
