# Supplementary figures and images for: Development of off-the-shelf hematopoietic stem cell-engineered invariant natural killer T cells for COVID-19 therapeutic intervention
Source: Stem Cell Res Ther. 2022 Mar 21;13:112. doi: 10.1186/s13287-022-02787-2 (PMC8935266; doi:10.1186/s13287-022-02787-2)

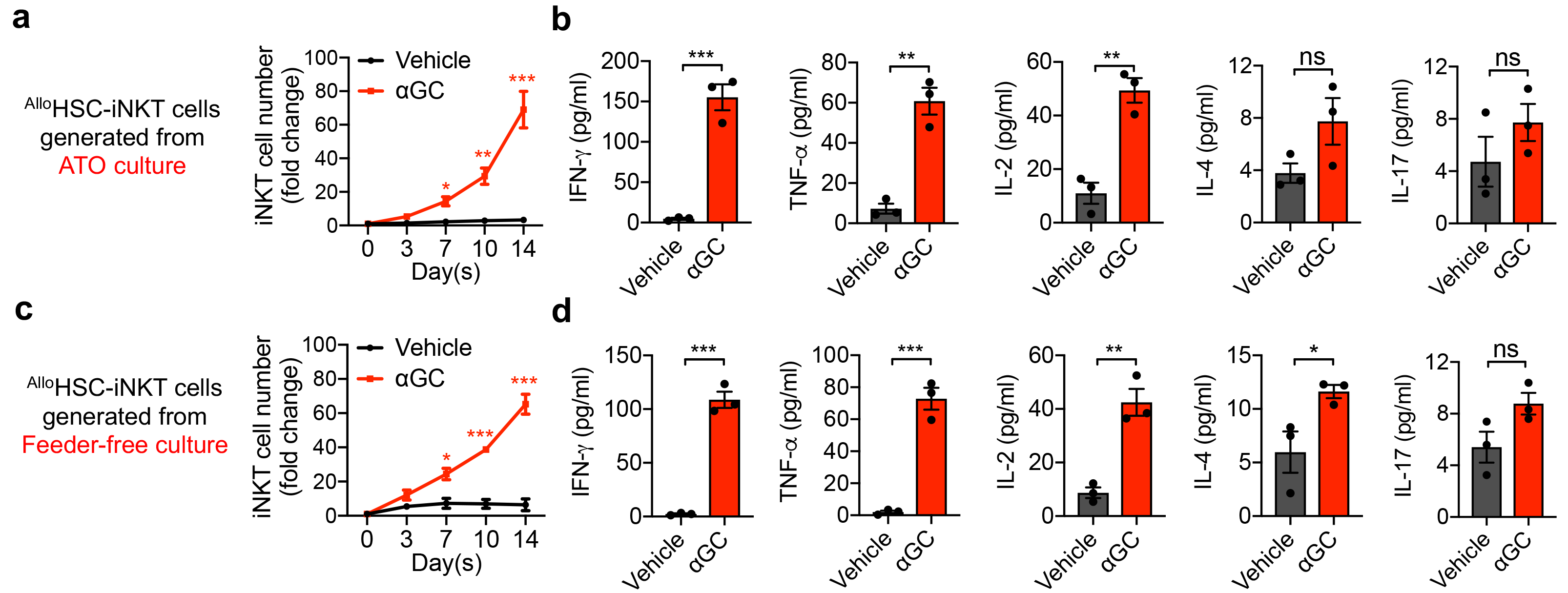

Supplement: Supplementary file 1 — Additional file 1. Fig. S1: Antigen responses of AlloHSC-iNKT cells; Related to Fig. 1. AlloHSC-iNKT cells generated from ATO and Feeder-free systems were cultured for 2 weeks, in the presence or absence of αGC (denoted as αGC or Vehicle, respectively). (a and c) Cell growth curve (n = 3). (b and d) ELISA analyses of cytokine (IFN-γ, TNF-α, IL-2, IL-4 and IL-17) production at day 7 post αGC stimulation (n = 3). [file 13287_2022_2787_MOESM1_ESM.tif]

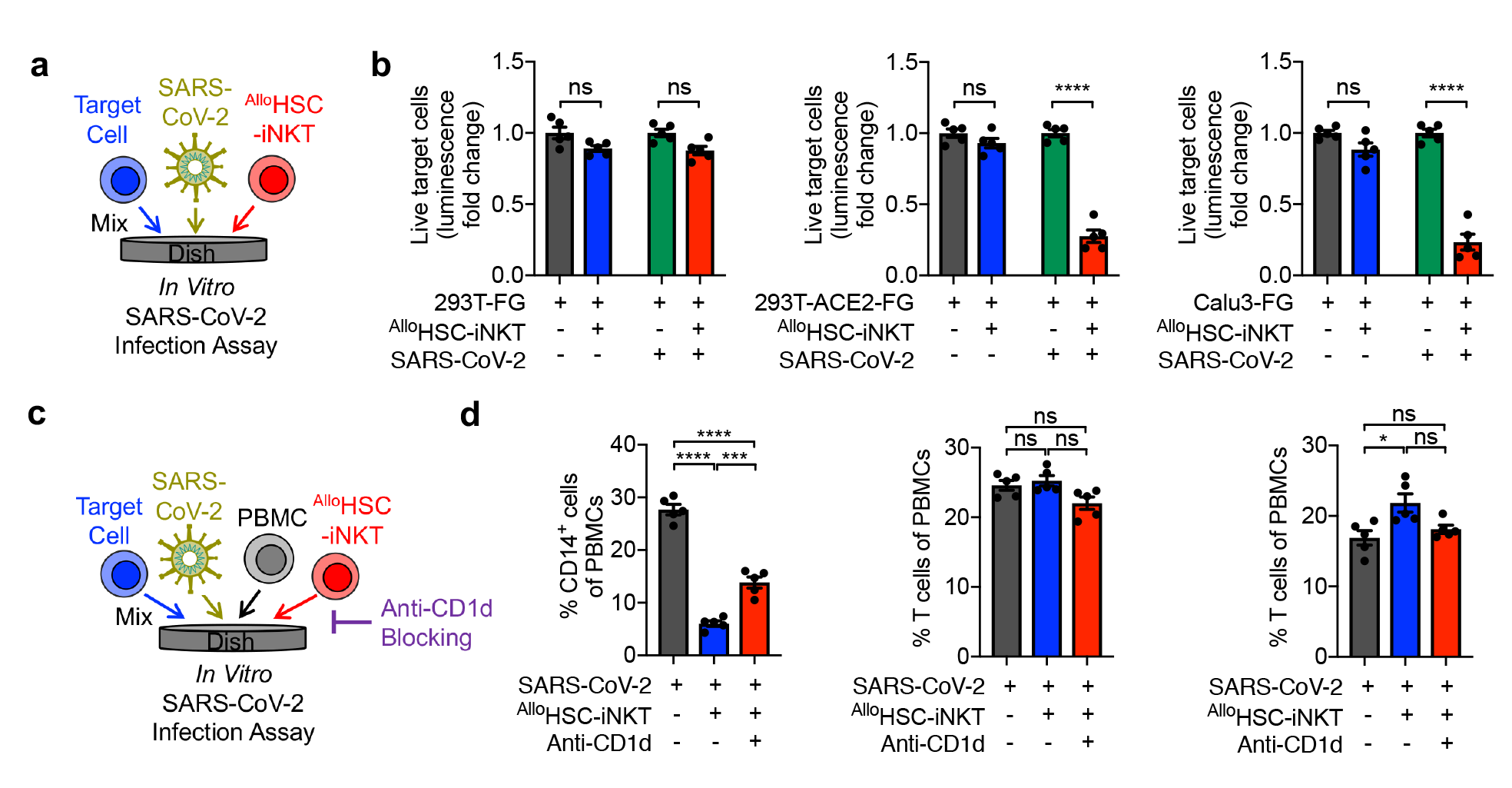

Supplement: Supplementary file 2 — Additional file 2. Fig. S2: Reduction of SARS-CoV-2 Virus Infection Load and Virus Infection-Induced Hyperinflammation by Feeder-Free Culture-Generated AlloHSC-iNKT Cells; Related to Fig. 2 and 3. (a-b) Study directly targeting of SARS-CoV-2 infected cells by Feeder-free culture-generated AlloHSC-iNKT cells. (a) Experimental design. (b) Target cell killing data of AlloHSC-iNKT cells at 24-h post co-culturing with infected cells (n = 5). (c-d) Study targeting virus-infection promoted inflammatory monocytes by Feeder-free culture-generated AlloHSC-iNKT cells. (c) Experimental design. (d) Flow cytometry analysis of remaining CD14+ monocytes, T and B cells in PBMCs after co-culturing with AlloHSC-iNKT cells. Representative of 3 experiments. Data are presented as the mean ± SEM. ns, not significant, *P < 0.05, **P < 0.01, ***P < 0.001, ****P < 0.0001, by Student's t test (b) and 1-way ANOVA (d). [file 13287_2022_2787_MOESM2_ESM.tif]

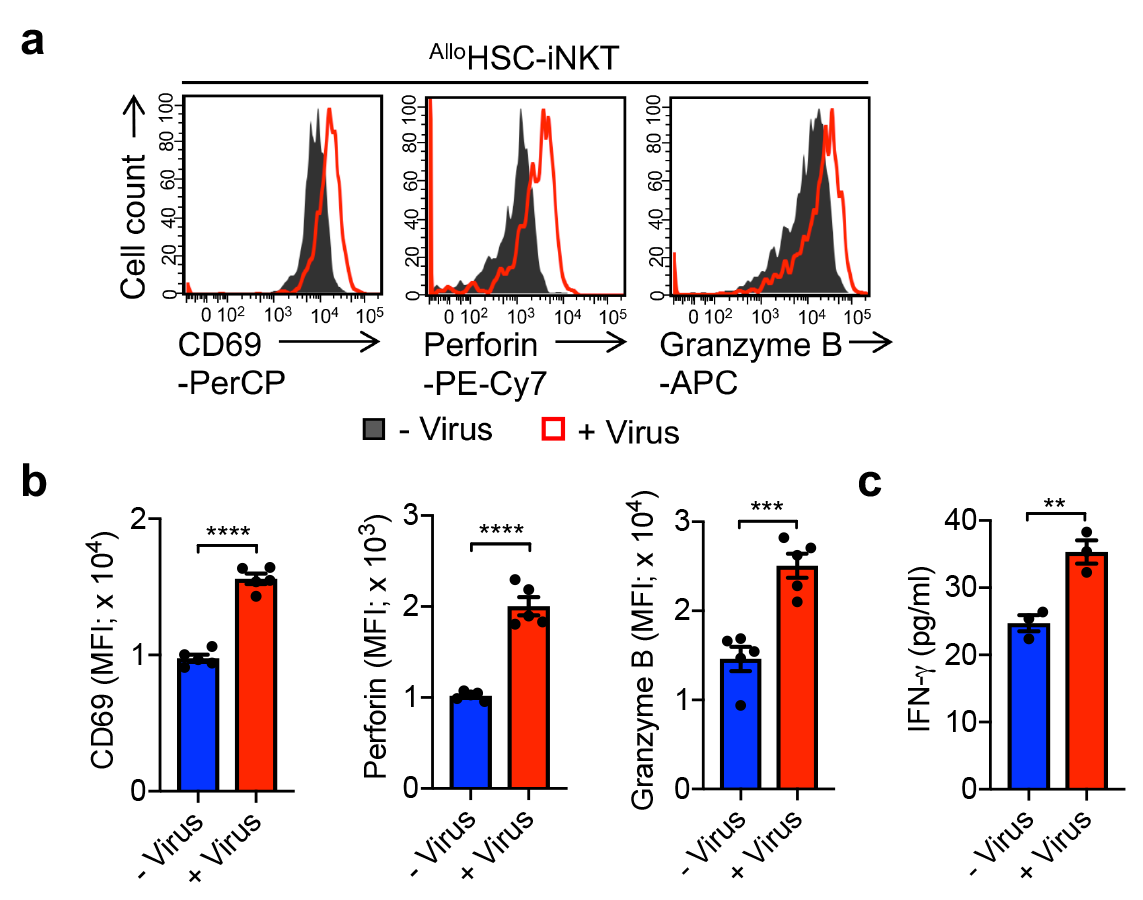

Supplement: Supplementary file 3 — Additional file 3. Fig. S3: AlloHSC-iNKT Cells are Activated by SARS-CoV-2 Infected Target Cells; Related to Fig. 2. (a) FACS detection of CD69, Perforin, and Granzyme B of AlloHSC-iNKT cells at 24-h post co-culturing with SARS-CoV-2 infected Calu3-FG cells. (b) Quantification of (a) (n = 5). (c) ELISA analysis of IFN-γ production (n = 3). Representative of 3 experiments. Data are presented as the mean ± SEM. ns, not significant, *P < 0.05, **P < 0.01, ***P < 0.001, ****P < 0.0001, by Student's t test. [file 13287_2022_2787_MOESM3_ESM.tif]

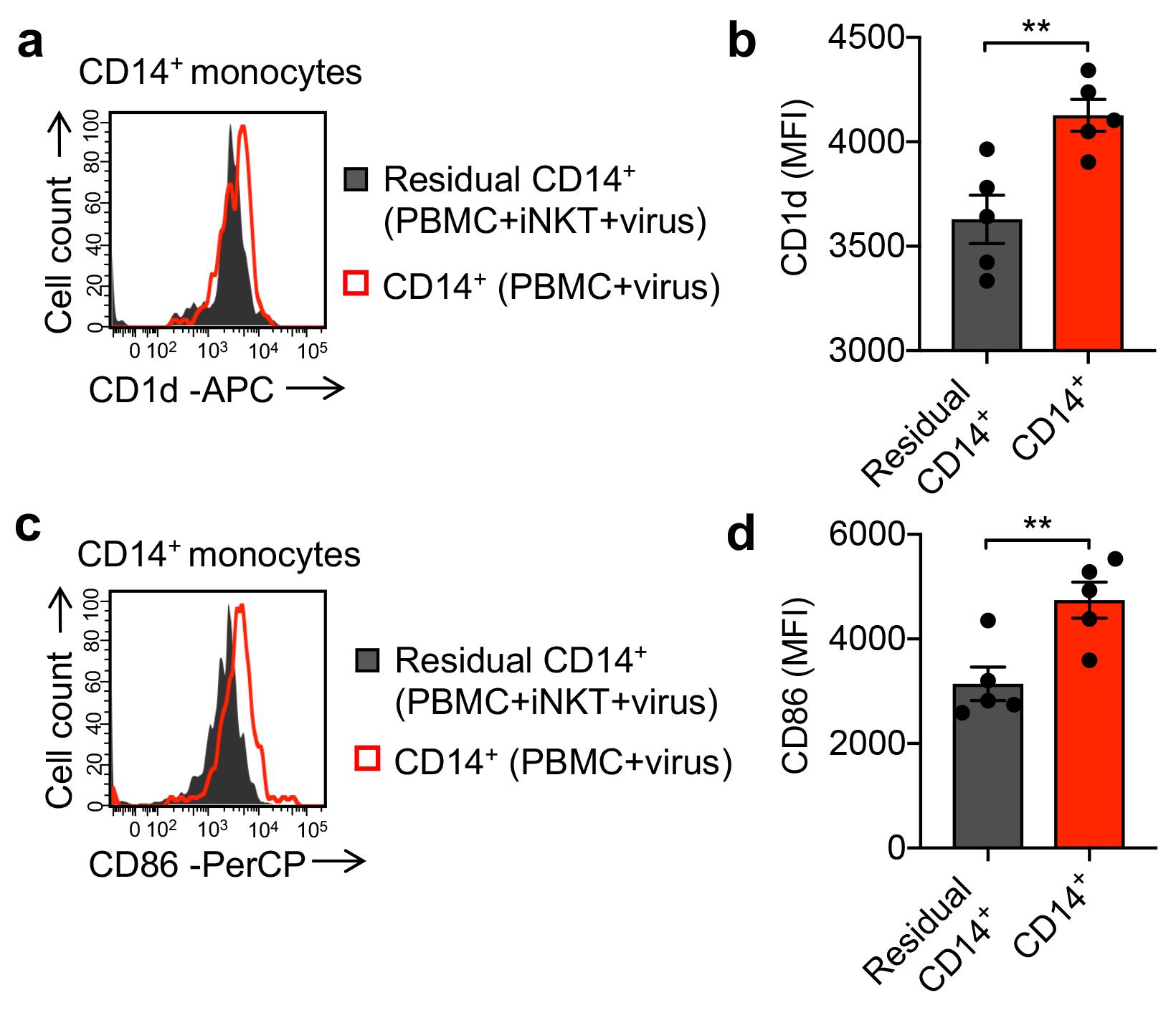

Supplement: Supplementary file 4 — Additional file 4. Fig. S4: Phenotype changes of CD14+ monocytes after co-culturing with AlloHSC-iNKT cells under SARS-CoV-2 infection; Related to Fig. 3. 293T-ACE2-FG cells were infected by SARS-CoV-2 virus. After 1 day, PBMCs were seeded in the culture with or without the addition of AlloHSC-iNKT cells and cultured for 24 h. Flow cytometry was used to detect cell populations. (a-b) FACS analyses of CD1d expression on CD14+ monocytes (n = 5). (c-d) FACS analyses of CD86 expression on CD14+ monocytes (n = 5). Data are presented as the mean ± SEM. **P < 0.01, by Student's t test. [file 13287_2022_2787_MOESM4_ESM.tif]
